# Complete Sequence and Annotation of the Mycoplasma phocicerebrale Strain 1049^T^ Genome

**DOI:** 10.1128/MRA.00514-19

**Published:** 2019-06-20

**Authors:** Salvatore Frasca, Jessy Castellanos Gell, Gerald F. Kutish, Dina L. Michaels, Daniel R. Brown

**Affiliations:** a Department of Comparative, Diagnostic, and Population Medicine, College of Veterinary Medicine, University of Florida, Gainesville, Florida, USA; b Department of Pathobiology and Veterinary Science and Center of Excellence for Vaccine Research, University of Connecticut, Storrs, Connecticut, USA; c Department of Infectious Diseases and Immunology, College of Veterinary Medicine, University of Florida, Gainesville, Florida, USA; Georgia Institute of Technology

## Abstract

The Mycoplasma phocicerebrale genome was analyzed to better understand this opportunistic pathogen. Amplification with ϕ29 polymerase was used to generate enough genomic DNA for large-insert library construction. Like other mycoplasmas from seals, *M. phocicerebrale* encodes an immunosuppressor that may predispose susceptibility to infection or influence intercurrent diseases of affected hosts.

## ANNOUNCEMENT

Mycoplasma phocicerebrale was first isolated from harbor seals (Phoca vitulina L.) that died along the North and Baltic seacoasts during a mass mortality event in 1988 (1, 2). To better understand its role in disease, we analyzed the genome of type strain 1049^T^ from the brain of a seal.

Two lyophilized aliquots of 1049^T^ broth culture were analyzed without further expansion. One had been deposited with the U.S. NIAID Mollicutes Collection (now part of World Data Centre for Microorganisms collection number 858; TMC) and the other with ATCC (catalog number 49640) in 1990 (1). The DNA was extracted from each specimen using an Easy-DNA kit (catalog number K180001; Thermo Fisher). The first specimen, from TMC, was prepared using the NEBNext Ultra TMII kit and dual-index multiplex oligos (catalog numbers E7645S and E7600S; New England Biolabs) for Illumina MiSeq 2 × 150-bp paired-end sequencing. Trimming and quality filtering with NxTrim v0.4.3 (3) yielded approximately 3 × 10^6^ Phred Q30 reads, but the draft *M. phocicerebrale* genome could not be closed using those data and the combination of PEAR v0.9.11, Ray v2.3.1, and Edena v3.131028 assembly software (4–6) or by PCR, indicating that a long-read strategy was needed. The second specimen, from ATCC, required amplification using REPLI-g ϕ29 polymerase (catalog number 150023; Qiagen) to generate enough genomic DNA for 20-kb insert library construction (Pacific Biosciences protocol 100-286-000). SEQUEL v6.0 single-molecule real-time sequencing yielded approximately 1 × 10^6^ uncorrected subreads (mean Q20 length, 9,427 bases; *N*_50_, 10,376 bases). Editing to remove chimeras or palindromes introduced by whole-genome amplification (7) was conducted using DASCRUBBER (https://github.com/rrwick/DASCRUBBER-wrapper). The quality-filtered Illumina and scrubbed SEQUEL data were combined to achieve a final *de novo* hybrid assembly also using Sprai v0.9.9.23 (http://zombie.cb.k.u-tokyo.ac.jp/sprai/index.html) and Canu v1.8r9332 (8), with postassembly corrections using MARVEL (https://github.com/schloi/MARVEL). The closed circular genome had 258× coverage depth and was annotated via RASTtk and NCBI Prokaryotic Genome Annotation Pipeline (PGAP) (9, 10). Default settings were used in all software.

This genome is 740,800 bp long with a GC content 25.0 mol%, which is close to the 25.9 mol% estimated by isopycnic centrifugation (1). It is interspersed with 10 IS*30* and 5 IS*150* elements. Two CRISPR arrays contain 16 and 8 spacer regions. Fewer than half of the 625 predicted open reading frames (ORFs) were assigned functions, and only one-fourth could be grouped in subsystems of structural RNAs; features of DNA, RNA, protein, carbohydrate, fatty acid, or phospholipid metabolism; transport or binding factors; and cell division factors. The genome amplification approach to library construction precluded inferences from the SEQUEL subreads regarding base methylation, but the annotation of cytosine 5-methyltransferase and type II restriction-modification methylase genes is evidence that the genome undergoes epigenetic modification.

*M. phocicerebrale* encodes one protein (DMC14_000785) with about 60% amino acid sequence similarity to the three orthologs of mycoplasmal protein M of Mycoplasma phocirhinis and about 40% similarity to the four orthologs of mycoplasmal protein M of Mycoplasma phocidae (11, 12). Protein M binds host IgG to block antigen-specific binding (13). No other determinants of tropism or virulence were discerned by genome analysis, although adherence and cytotoxicity of *M. phocicerebrale* to epithelial cells has been demonstrated *in vitro* (14). Immunosuppression may constitute a mechanism that enables *M. phocicerebrale* to evade host defenses or modulate intercurrent diseases of affected individuals (15–18).

### Data availability.

The *M. phocicerebrale* 1049^T^ genome sequence and annotation have been deposited in GenBank under the accession number CP033058 and assembly accession number GCA_003383595; the assembly described in this paper is the latest version, ASM338359v3. The raw data are available in the NCBI Sequence Read Archive linked to BioProject accession number PRJNA473817.
